# Cushing's syndrome in pregnancy in which laparoscopic adrenalectomy was safely performed by a retroperitoneal approach

**DOI:** 10.1002/iju5.12637

**Published:** 2023-09-10

**Authors:** Nobuyoshi Takeuchi, Yusuke Imamura, Kazuki Ishiwata, Manato Kanesaka, Yusuke Goto, Tomokazu Sazuka, Sawako Suzuki, Hisashi Koide, Shinichi Sakamoto, Tomohiko Ichikawa

**Affiliations:** ^1^ Department of Urology Chiba University Graduate School of Medicine Chiba Japan; ^2^ Department of Clinical Cell Biology Chiba University Graduate School of Medicine Chiba Japan

**Keywords:** adrenalectomy, Cushing's syndrome, pregnancy, retroperitoneal approach

## Abstract

**Introduction:**

Laparoscopic adrenalectomy is the standard treatment for adrenal tumors caused by Cushing's syndrome. However, few pregnant women have undergone adrenalectomy because of the risk of general anesthesia and surgery.

**Case presentation:**

A 28‐year‐old woman presented with gradually worsening Cushing's signs at around 12 weeks of pregnancy. Magnetic resonance imaging displayed a 38‐mm left adrenal tumor, which was the cause of the adrenal Cushing's syndrome. Metyrapone was started, which increased androgen levels. Since the management of Cushing's syndrome by medication alone is challenging, unilateral laparoscopic adrenalectomy by a retroperitoneal approach was performed at 23 weeks of the pregnancy. No perioperative complications were noted.

**Conclusion:**

Adrenalectomy is considered safe in pregnant women with Cushing's syndrome. Laparoscopic adrenalectomy by retroperitoneal approach should be chosen and performed between 14 and 30 weeks of pregnancy to prevent mother and fetal complications.

**Abbreviations & Acronyms**
CS: Cushing's syndrome
MRI: magnetic resonance imaging

**Keynote message**

We report a rare case of adrenalectomy performed via a retroperitoneal approach for Cushing's syndrome in a pregnant woman. Cushing's syndrome may affect the fetus, and surgery can be considered in addition to medical management. Adrenalectomy should be performed in the second trimester of pregnancy. Pneumoperitoneal pressure, position, and surgical approaches must receive careful attention.

## Introduction

CS is characterized by excessive cortisol secretion and characteristic symptoms such as full moon‐like facial features and central obesity. Premenopausal women with CS rarely become pregnant because excessive glucocorticoid secretion inhibits the synthesis of gonadotropins, leading to impaired ovarian and endometrial function, and causing amenorrhea or oligomenorrhea.^1^ Furthermore, even when women with CS become pregnant, the incidence of severe complications is high. CS can cause maternal hypertension, diabetes/glucose intolerance, osteopenia/osteoporosis, preeclampsia, pulmonary edema, heart failure, opportunistic infections, and even death. Additionally, CS can potentially cause stillbirth, prematurity, and intrauterine fetal growth restriction.^1^, ^2^, ^3^, ^4^, ^5^, ^6^ Therefore, CS must be detected at an early stage in pregnancy; however, CS may go undetected because of the overlapping signs of preeclampsia and/or gestational diabetes.

A cortisol‐secreting adrenal tumor is the underlying cause of CS, and laparoscopic adrenalectomy is the standard treatment to it. Medical treatment of CS can include medications that inhibit 11β‐hydroxylase, such as metyrapone and osilodrostat, but surgical treatment is considered if the disease is difficult to control with medical treatment. Nonobstetric surgery during pregnancy is performed in 1%–2% of pregnant women.^7^ Although general anesthesia is relatively safe during pregnancy, the indication for the surgery must be carefully considered because of potential risks such as neurodevelopmental delay, sudden death, etc.

Herein, we present a case of a pregnant woman diagnosed with CS who underwent unilateral laparoscopic adrenalectomy by a retroperitoneal approach without any problems.

## Case presentation

The patient was a 28‐year‐old primiparous woman. Since around 12 weeks of pregnancy, she has experienced facial and lower limb edema; gained 6‐kg weight in 1 month; increased facial acne; and experienced subcutaneous bleeding on the forearms, red abdominal dermatitis, proximal muscle weakness, palpitations, insomnia, and decreased vision in eyes. Her symptoms gradually worsened from 14 weeks, and she was referred to our hospital to clarify the cause at 18 weeks of pregnancy.

**Fig. 1:**
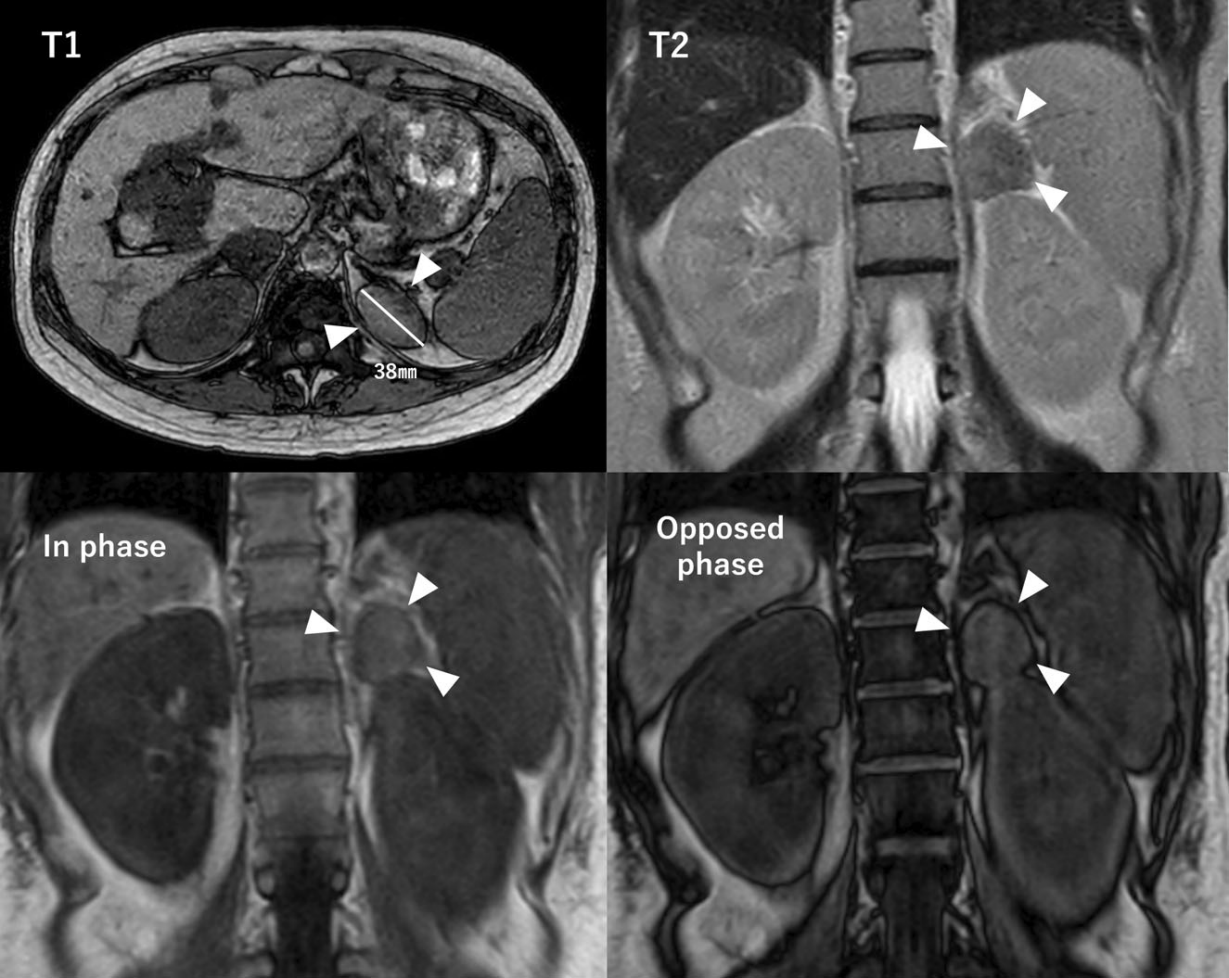
Magnetic resonance imaging on admission shows a left adrenal tumor with a long axis of 38 mm (arrowhead). Signal reduction was partially observed on opposed‐phase images, leading to diagnosis of cortical adenoma.

Adrenal CS was suspected on the basis of her Cushing's signs, cortisol 25 μg/dL, and adrenocorticotropic hormone <1.5 pg/mL. She had hypokalemia, hypogammaglobulinemia, and liver dysfunction, and her condition was rapidly worsening. Given her pregnant state, she was admitted for intensive testing for the case of CS from 19 weeks of pregnancy. MRI revealed a well‐defined 38‐mm left adrenal tumor, which was the cause of the adrenal CS (Fig. 1). She was started on metyrapone with 250 mg per day, which increased androgens (0.53–0.69 ng/mL in 1 week). We considered that the management of CS by medication alone would be challenging and performed adrenalectomy during her pregnancy. The dose of metyrapone was increased to 1000 mg per day eventually.

**Fig. 2:**
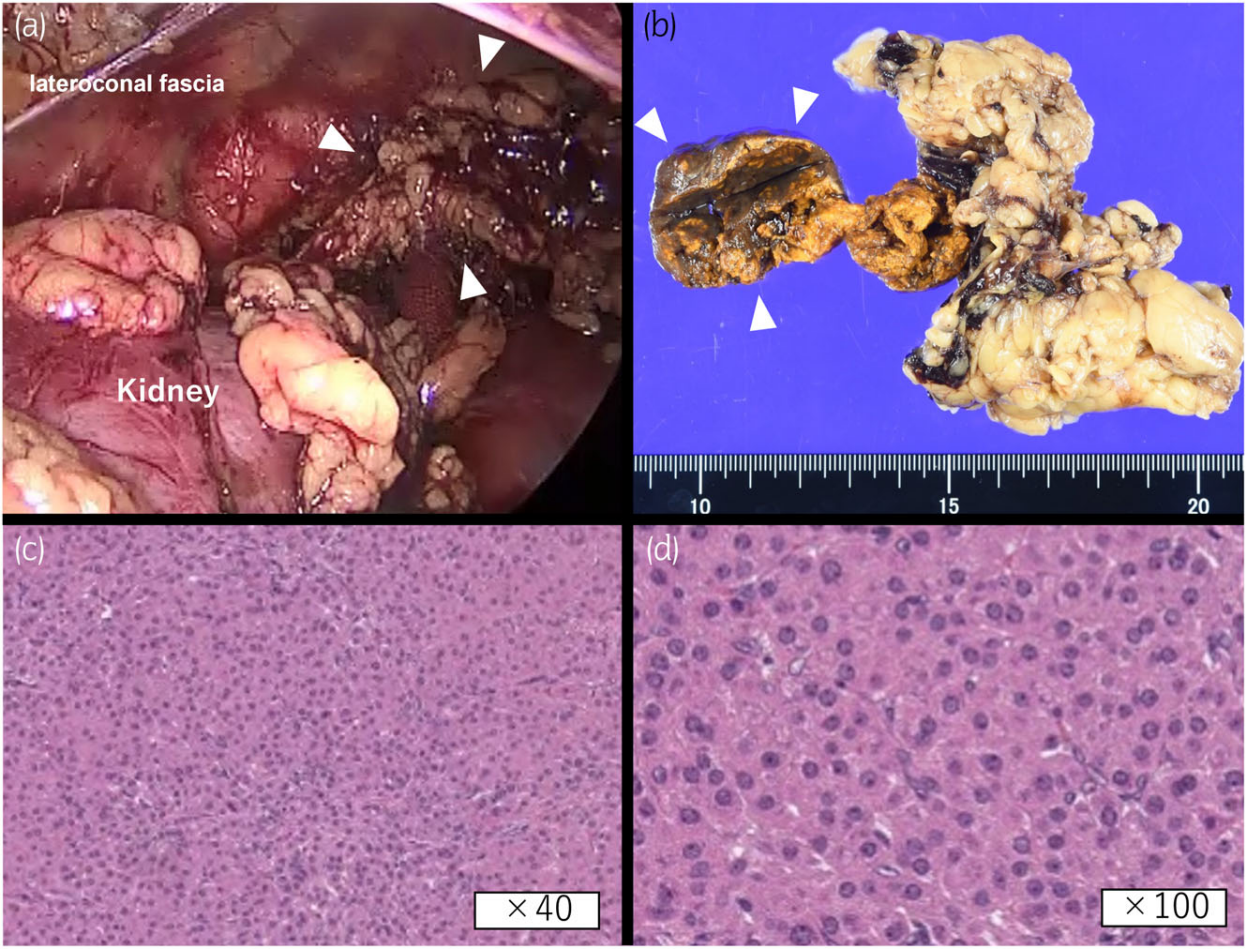
(a) Intraoperative findings of the retroperitoneal approach. Arrowheads indicate the tumor. (b) Gross appearance of the resected adrenal tumor; a brownish‐toned, substantial mass, 60 × 34 × 15 mm in size. (c, d) Hematoxylin–eosin staining showed that nodular lesion with a fibrous capsule, with foci of homogeneous cells with eosinophilic or pale, foamy sporangia and small round nuclei.

She was admitted to the hospital at 23 weeks and 2 days of gestation, and laparoscopic left adrenalectomy was performed via a retroperitoneal approach in the right lateral and jackknife position on the following day (Fig. S1). During the surgery, blood pressure was carefully controlled by an anesthesiologist and the patient's position and fetal heart rate were monitored by an obstetrician. The operation time, insufflation time, and general anesthesia time were 68, 59, and 123 min, respectively, and the blood loss volume was 75 mL, without any complications. Pathological findings revealed an adrenocortical adenoma. The specimen was positive for one of the nine Weiss criteria (Fig. 2).

Postoperatively, metyrapone was discontinued and both lower leg edema, facial acne, fatigue, and muscle weakness improved. Metyrapone was discontinued after surgery. Hydrocortisone, which had been administered at 150 mg/day during the perioperative period, was reduced every few weeks and was taken at 30 mg/day at delivery. She delivered by cesarean section at 38 weeks and 2 days of gestation, with good outcomes for the mother and her infant. Hydrocortisone was discontinued 15 weeks after delivery.

**Fig. 3:**
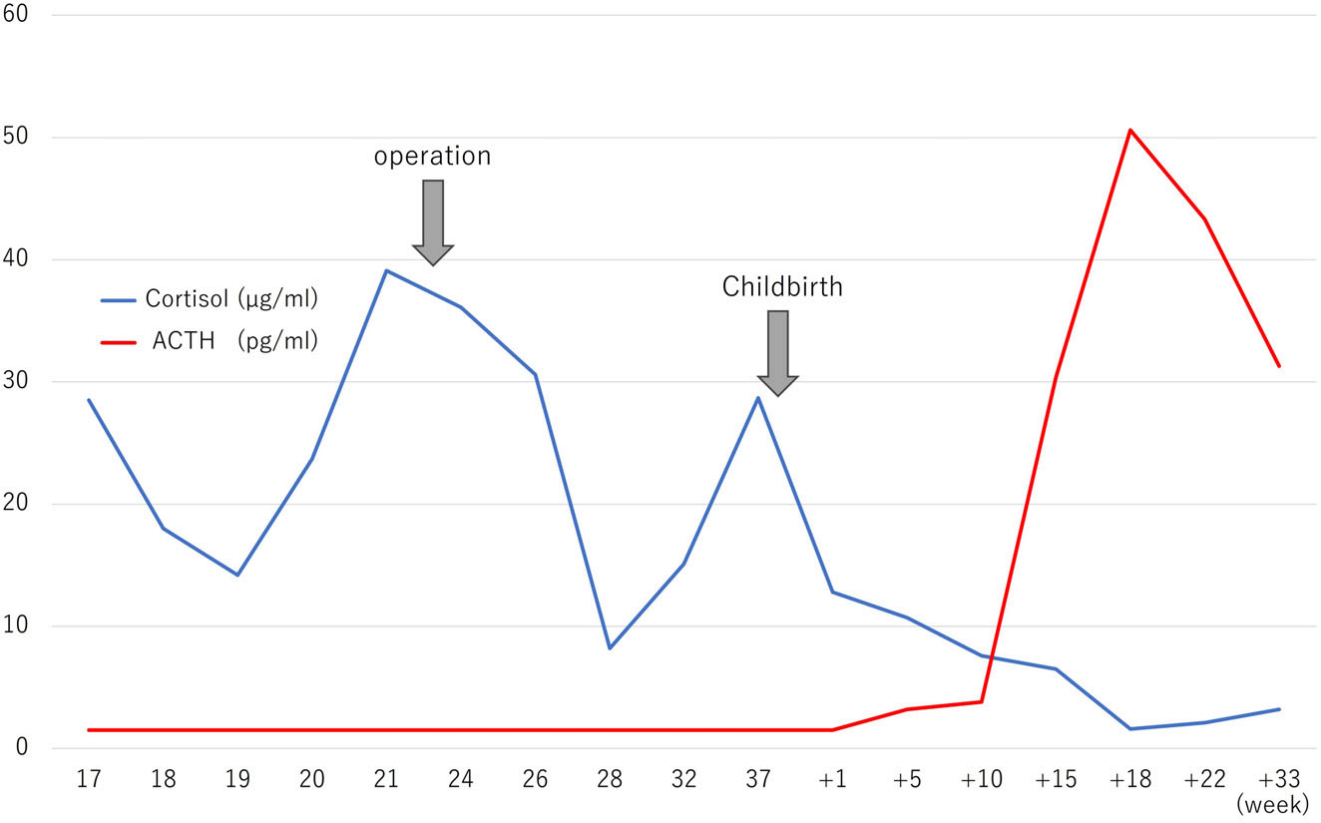
The transition of Cortisol and ACTH. Cortisol decreases rapidly after surgery and rises again before delivery. As cortisol improved, ACTH also increased.

We showed the changes in cortisol and ACTH from the first visit to postpartum (Fig. 3).

## Discussion

CS seldom occurs during pregnancy. Symptoms such as weight gain, skin striae, fatigue, and a round face can also occur in normal pregnancies. The dexamethasone suppression test can result in false positives because of ACTH produced by placenta in normal pregnancy. During pregnancy, there is a physiological state of high cortisol levels. The disappearance of diurnal rhythm is a useful indicator for diagnosis of CS in pregnancy because circadian rhythm is maintained in normal pregnancy. Useful diagnostic criteria include urine cortisol levels greater than three times the upper limit of normal, loss of diurnal cortisol rhythm, and presence of adrenal tumors on MRI.

The pharmacologic treatment of endogenous cortisol is complex, and hormonal management is challenging. While the management of the cortisol levels is important, metyrapone is a risk factor for gestational hypertension and may inhibit fetal cortisol production by crossing the placenta.^1^, ^2^, ^3^, ^4^, ^5^, ^6^, ^8^, ^9^, ^10^, ^11^, ^12^

In this case, because androgens were also elevated and drug management was expected to be challenging, the surgery was aggressively considered. Despite the reports of successful adrenalectomy is after 28 weeks of gestation,^6^, ^13^, ^14^ The surgery should be performed by an experienced team between 14 and 30 weeks of pregnancy, that is, after organogenesis phase and before the fetus grows too large.^1^, ^13^, ^15^

A few pregnant women with adrenal CS undergo adrenalectomy. However, the laparoscopic approach is safe, and maternal and fetal complications were higher in women who did not undergo surgery.^16^ Less postoperative pain, faster wound healing, and faster postoperative recovery are the main advantages of laparoscopic surgery.^17^

In pregnant women, pneumoperitoneal pressure should be kept <12 mmHg because increased intraabdominal pressure decreases placental blood flow and can cause fetal acidosis due to the absorption of carbon dioxide used for insufflation.

Laparoscopic adrenalectomy can be safely performed through both transperitoneal and retroperitoneal approaches.^18^ However, in pregnant women, performing the surgery by the retroperitoneal approach in the lateral position is preferable to prevent putting pressure on the fetus during the surgery. The retroperitoneal approach is advantageous, as less pressure is placed on the uterus and adhesions are prevented. After taking the lateral position, the obstetrician is advised to check the position and confirm that the abdomen is not compressed and that the fetal heart rate is normal.

## Conclusions

We present a case of a pregnant woman diagnosed with adrenal CS who underwent a unilateral laparoscopic adrenalectomy by a retroperitoneal approach without any problems. Adrenalectomy is a useful treatment when CS is difficult to control despite metyrapone and other medical support.

## Author contributions

Nobuyoshi Takeuchi: Conceptualization; methodology; project administration; writing – original draft. Yusuke Imamura: Conceptualization; methodology; supervision; writing – review and editing. Kazuki Ishiwata: Data curation; supervision. Manato Kanesaka: Data curation; supervision. Yusuke Goto: Data curation; supervision. Tomokazu Sazuka: Data curation; supervision. Sawako Suzuki: Data curation; supervision. Hisashi Koide: Data curation; supervision. Shinichi Sakamoto: Data curation; supervision. Tomohiko Ichikawa: Data curation; supervision.

## Conflict of interest

The authors declare no conflicts of interest.

## Approval of the research protocol by an Institutional Reviewer Board

Not applicable.

## Informed consent

Informed consent for the release of the case report and accompanying images has been obtained from the patient.

## Registry and the Registration No. of the study/trial

Not applicable.

## Supporting information


**Figure S1.** The illustration of port placement. We used 12 mm camera port, 12 and 5 mm port for forceps.
