# Supplementary figures and images for: Cushing's syndrome in pregnancy in which laparoscopic adrenalectomy was safely performed by a retroperitoneal approach
Source: IJU Case Rep. 2023 Sep 10;6(6):415–8. doi: 10.1002/iju5.12637 (PMC10622216; doi:10.1002/iju5.12637)

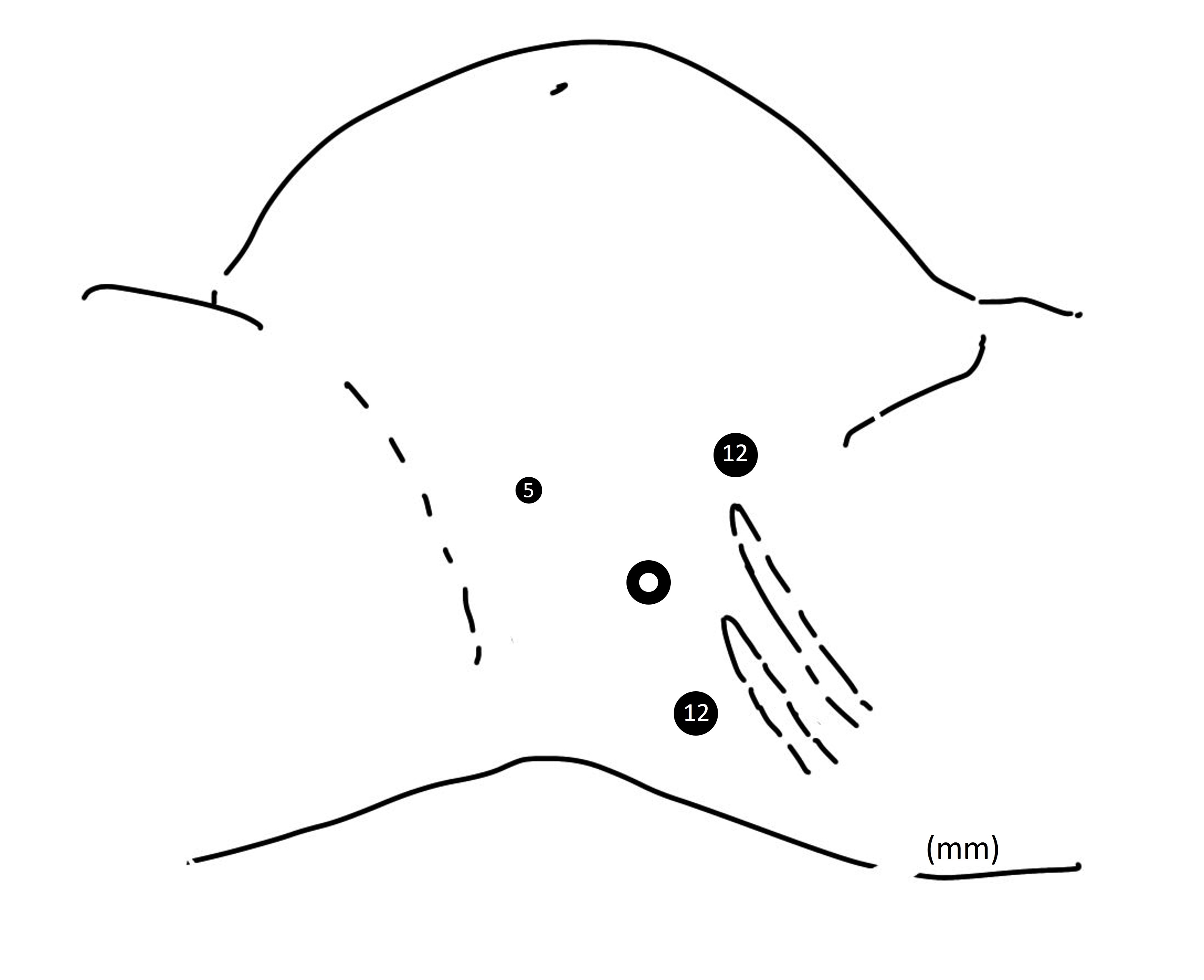

Supplement: Supplementary file 1 — Figure S1. The illustration of port placement. We used 12 mm camera port, 12 and 5 mm port for forceps. [file IJU5-6-415-s001.jpg]
